# Noneffect of SARS-CoV-2 spike glycoprotein Y217N mutation on affinity between the virus and ACE2

**DOI:** 10.1016/j.jbc.2021.100725

**Published:** 2021-05-27

**Authors:** Takuma Hayashi, Ikuo Konishi

**Affiliations:** 1 National Hospital Organization, Kyoto Medical Center, Kyoto, Japan; 2 Japan Science and Technology Agency (JST) START-Program Project Team, Tokyo, Japan; 3 Department of Obstetrics and gynecology, Kyoto University Graduate School of Medicine, Kyoto, Japan

The interaction between the severe acute respiratory syndrome coronavirus 2 (SARS-CoV-2) and angiotensin-converting enzyme 2 (ACE2), the primary entry receptor for SARS-CoV-2, is a key determinant of the range of hosts that can be infected by the virus. Zhang *et al.* (1) reportedly constructed human ACE2 (hACE2) with the Y217N mutation and found that this mutation completely blocked SARS-CoV-2 entry. Zhang *et al.* (1) performed an receptor binding domain (RBD) binding assay and found that WT hACE2 potently bound the RBD; however, hACE2 Y217N almost lost the ability to bind the RBD.

Our experiment using Spanner, a structural homology modeling pipeline method, revealed that the three-dimensional structure of the binding region containing the investigated five amino acids, 353-KGDFR-357 of hACE2 N217 involved in binding interactions with the spike glycoprotein of SARS-CoV-2, was completely conserved in comparison with the structure of hACE2 Y217.

Structural remodeling analyses using the PDBePISA tool and MOE project DB (MOLSIS Inc) also suggested that the Y217N substitution in amino acid residues in the subdomain II motif of hACE2 (2) did not significantly decrease the binding affinity of the RBD of SARS-CoV-2 (Fig. 1, Appendix). As the binding free energy did not change, the affinity between the ligand and receptor is fairly constant. No reports show the essential of the amino acid residues of the subdomain II motif of hACE2 for the binding of ACE2 to RBD (Appendix) (2, 3).

**Figure 1:**
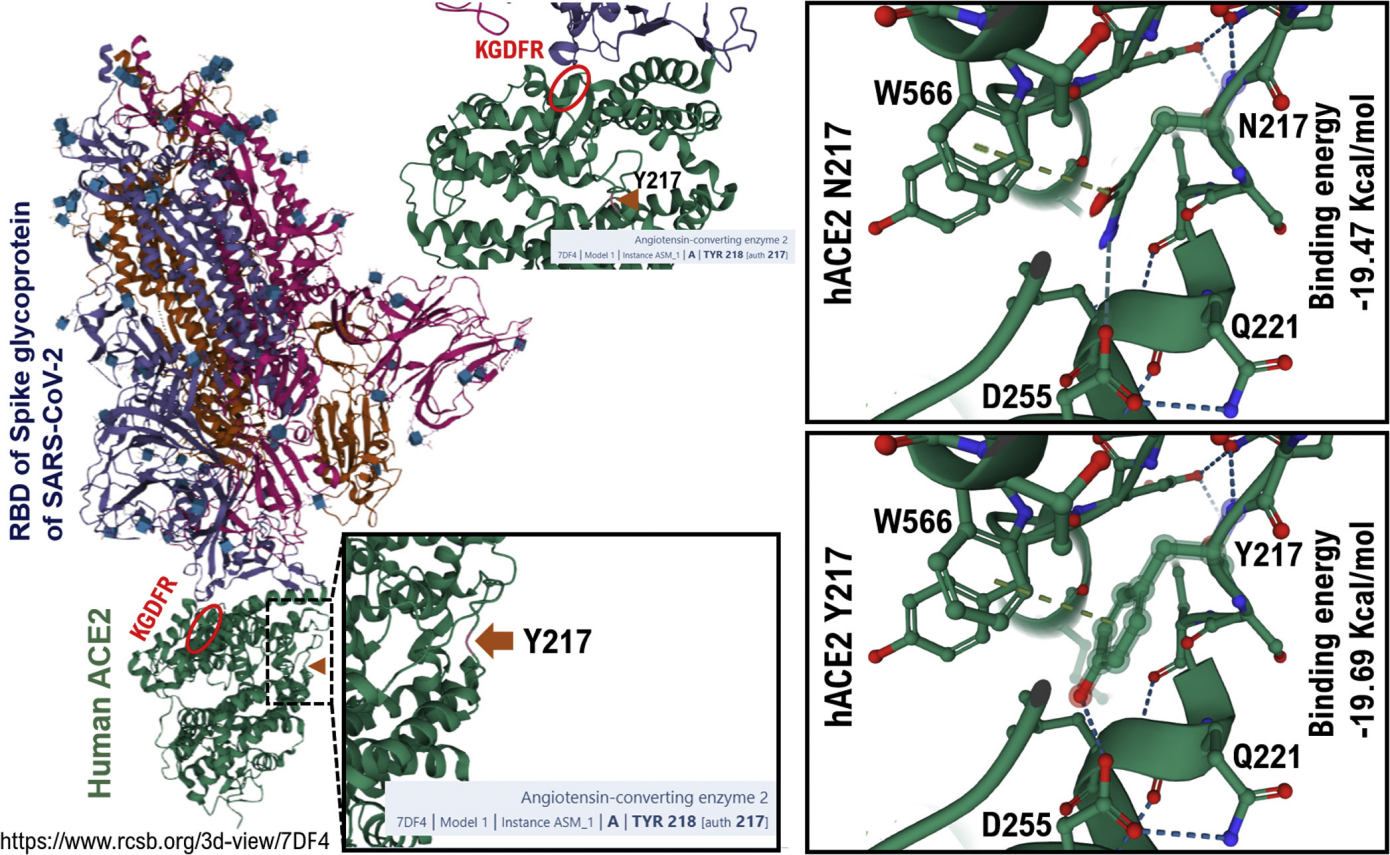
**The complex structure of RBD of spike glycoprotein of SARS-CoV-2 bound to human ACE2.** A cartoon representation of the complex structure is analyzed using the LigPlot + program (v.1.4.5) and MOE project DB (MOLSIS Inc). The core and external subdomains in RBD in the spike glycoprotein of SARS-CoV-2 are colored in *blue purple*. Human ACE2 (hACE2) subdomains I and II are *green*, respectively. Key contact sites are marked with the two right *boxes* in three-dimensional structures and are further delineated for interaction details, respectively. The homology modeling of hACE2 with SARS-CoV-2 RBD with Y217 (*lower right*) or N217 (*upper right*) residue is reported. Protein buried surface areas are analyzed using the PDBePISA tool and MOE project DB (MOLSIS Inc). As the binding free energy did not change, the affinity between ACE2 and RBD of spike glycoprotein of SARS-CoV-2 is fairly constant. RBD, receptor binding domain; SARS-CoV-2, severe acute respiratory syndrome coronavirus 2.

Overall, the virus–receptor engagement is dominated by polar contacts mediated by the hydrophilic residues (4). In support of this hypothesis, a single Y217N substitution was sufficient to conserve these interactions.

## Supporting information

This article contains supporting information.

## Conflict of interest

The author declares no conflicts of interests with the contents of this article.
